# Progress in research on gut microbiota regulation of tumorigenesis, progression, and therapeutic response via inflammatory cytokines

**DOI:** 10.3389/fimmu.2026.1922678

**Published:** 2026-10-12

**Authors:** Yan Liu, Dan Zang, Jun Chen

**Affiliations:** Department of Oncology, The Second Hospital of Dalian Medical University, Dalian, China

**Keywords:** cancer therapy, chronic inflammation, gut microbiota, inflammatory cytokines, tumorigenesis and progression

## Abstract

The gut microbiota has emerged as an important regulator of tumour biology through dynamic interactions with host immune and inflammatory networks. A growing number of preclinical studies and observational clinical studies suggest that dysbiosis-associated changes in microbial composition and metabolite profiles can remodel inflammatory signalling pathways and are associated with tumour initiation, progression, metastasis, and therapeutic response. Microbial components and metabolites can activate pattern-recognition receptor-related pathways, including nuclear factor kappa B (NF-κB), in cell and animal models. These changes may promote a tumour-supportive chronic inflammatory milieu or, under specific ecological conditions, help shape an immune-activated microenvironment. Current evidence also suggests that the gut microbiota and inflammatory cytokines may influence treatment response. Preclinical models and some patient cohort studies indicate that dysregulated inflammatory responses are associated with reduced chemotherapy tolerance and a higher risk of immune-related adverse events (irAEs), whereas certain beneficial microbial signals are associated with enhanced CD8^+^ T-cell activation and improved efficacy of immune checkpoint inhibitors (ICIs). However, these associations vary across cancer types and study populations, and their causality and clinical generalizability still require further validation. In this Review, we summarize current evidence on how the gut microbiota and inflammatory cytokines jointly regulate tumour development and therapeutic outcomes. We also discuss therapeutic strategies that target microbial composition and inflammatory signalling, with emphasis on their translational potential in precision oncology.

## 1 Introduction

Tumorigenesis is a multistep process driven by genetic, environmental, immune, and inflammatory factors. Chronic inflammation is widely recognized as a key component of cancer development and progression (1). Many studies have shown that chronic inflammation not only promotes genetic mutations in tumour cells (2), but also remodels the tumour microenvironment (TME), making it more permissive for tumour cell survival, proliferation, invasion, and immune evasion (3).

Inflammatory homeostasis is maintained by a balance between pro-inflammatory and anti-inflammatory mediators. Inflammatory cytokines are small secreted proteins produced by immune cells such as macrophages and T cells, as well as by epithelial cells and fibroblasts. As important members of the cytokine family, they mediate intercellular communication and link the immune system to the tissue microenvironment. Therefore, they play complex and central roles in tumour biology (4). Pro-inflammatory cytokines include tumour necrosis factor-α (TNF-α), interleukin-1β (IL-1β), IL-6, IL-8, IL-12, IL-17, and interferon-γ (IFN-γ). These cytokines initiate and amplify inflammatory responses and recruit immune cells (5). Anti-inflammatory cytokines include IL-4, IL-10 (6), and transforming growth factor-β (TGF-β) (7). These mainly suppress the production of pro-inflammatory mediators.

During inflammation-driven tumorigenesis, the gut microbiota has an important role in maintaining physiological homeostasis and triggering pathological inflammation by regulating host immune and metabolic balance. The human gut contains approximately 101410^{14}1014 microorganisms, including thousands of bacterial taxa, and its total gene content exceeds that of the human genome by more than 100-fold (8). This complex ecosystem is closely linked to the host immune system, metabolic state, and inflammatory responses. Under physiological conditions, a balanced gut microbiota helps maintain immune tolerance through its metabolites and cellular components, promotes regulatory T-cell (Treg) differentiation and anti-inflammatory cytokine secretion, and creates an immune microenvironment that is less favourable for tumour growth (9). When the composition and function of the gut microbiota become dysregulated, this balance is disrupted. This disruption can impair the local intestinal barrier and promote systemic inflammatory responses (10) and immune abnormalities (11). Increasing evidence suggests that gut microbiota dysbiosis is an important risk factor for several malignancies.

In recent years, advances in metagenomic sequencing, metabolomics, and single-cell immunomics have shown that the gut microbiota regulates inflammatory cytokines through multiple pathways and participates in tumour initiation, promotion, and metastasis. In this way, it can influence tumour occurrence and progression. However, many existing reviews focus mainly on how the gut microbiota affects immunotherapy response. In contrast, the bidirectional regulation between the microbiota and inflammatory cytokines during standard anti-cancer treatment, and the translation of these mechanisms into clinically actionable interventions, remain less well integrated. This narrative review summarizes current evidence on the role of the gut microbiota and its metabolites in treatment response and prognosis, particularly in non-small cell lung cancer (NSCLC). It has three main aims. First, this review summarizes the direct effects of standard chemotherapy and immunotherapy on gut microbiota composition and metabolite production in common solid tumours, including NSCLC. Second, it analyses how the microbiota and its metabolites bidirectionally regulate therapeutic efficacy and toxicity through inflammatory cytokine networks. Third, it integrates current microbiota-based intervention strategies and their translational potential in clinical trials, with the goal of identifying feasible paths toward precision microbiota-based therapy.

## 2 Mechanisms by which the gut microbiota and its metabolites regulate tumour development via inflammatory cytokines

Tumour development can be broadly divided into three stages: tumour initiation, tumour promotion, and tumour progression (12). The gut microbiota may regulate each stage through inflammatory cytokines and related signalling pathways. The toll-like receptor 4/myeloid differentiation primary response 88 (TLR4/MyD88) pathway mainly mediates recognition of microbial-associated molecular patterns such as lipopolysaccharide (LPS) and initiates innate immune inflammatory responses. Nuclear factor kappa B (NF-κB) is a key transcriptional hub that integrates multiple pro-inflammatory signals and induces expression of cytokines such as TNF-α, IL-1β, and IL-6. Signal transducer and activator of transcription 3 (STAT3) often acts as a downstream effector of inflammatory cytokines and metabolic signals and participates in cell survival, proliferation, immunosuppression, and angiogenesis. Overall, these effects can be grouped into three major mechanisms. First, microbial structural components activate pattern-recognition receptors and downstream inflammatory signalling. Second, microbial metabolites regulate host immunity and tumour behaviour through metabolism–inflammation coupling. Third, sustained interactions between the microbiota and inflammatory networks remodel the tumour immune microenvironment at the systemic level.

### 2.1 Microbial structural components activate inflammatory signalling through pattern-recognition receptors

Under healthy conditions, the gut microbiota helps suppress carcinogenesis by maintaining barrier integrity and mucosal immune homeostasis, promoting Treg differentiation, and supporting anti-inflammatory cytokine secretion. When this balance is disrupted and intestinal mucosal barrier function declines, bacteria and their structural components can more easily cross the epithelial barrier, induce inflammatory responses, and promote tumorigenesis.

At the stage of tumour initiation, preclinical models suggest that gut microbiota dysbiosis promotes inflammation and increases recruitment of pro-tumorigenic neutrophils (13). When microbial diversity decreases or the relative abundance of opportunistic pathogens increases, structural components such as LPS, lipoteichoic acid, flagellin, and peptidoglycan can cross the damaged intestinal epithelial barrier more easily and activate pattern-recognition receptor-associated inflammatory pathways. Animal studies have shown (14) that imbalanced gut microbial diversity can induce pro-inflammatory responses in the liver, aggravate inflammation and fibrosis, and eventually promote hepatocarcinogenesis. In cell and animal experiments, outer membrane vesicles secreted by Fusobacterium nucleatum can be taken up by intestinal epithelial cells and activate intracellular inflammatory transcriptional programmes. This process leads to markedly increased messenger RNA (mRNA) expression of KC, the murine homologue of IL-8, TNF-α, IL-6, IFN-γ, and monocyte chemoattractant protein-1 (MCP-1) in colonic mucosal tissue, suggesting that this bacterium can disrupt intestinal homeostasis and drive local pro-inflammatory responses in an outer membrane vesicle-dependent manner (15). In addition, toxins from Clostridioides difficile can promote IL-1β production (16), bacterial peptidoglycan can induce pro-inflammatory signalling (17), and flagellin can activate inflammasomes and induce pro-inflammatory cytokine production (18). These inflammatory stimuli may further induce oxidative stress and DNA damage (19), thereby promoting the accumulation of genetic alterations and facilitating tumorigenesis.

At the stage of tumour promotion, gut microbiota dysbiosis causes persistent barrier disruption (20). This disruption allows bacterial products to continuously enter the circulation, activate immune cells, and maintain a chronic pro-inflammatory state. Analysis of fresh faecal samples from mice with oesophageal cancer showed that an increased abundance of the phylum Bacteroidetes was associated with the development of oesophageal squamous cell carcinoma (ESCC). LPS derived from these bacteria entered the circulation and triggered a typical pro-inflammatory transcriptional response, with increased IL-6, TNF-α, and TGF-β and reduced IL-10, thereby inducing systemic inflammation and promoting ESCC invasion (21). TNF-α can further increase intestinal mucosal permeability (22), allowing bacteria to cross the epithelial barrier more readily and enter the bloodstream, which may amplify systemic inflammatory responses. Previous studies have shown that IL-1 family cytokines such as IL-18 and IL-1α are activated under dysbiotic conditions and can promote increased intestinal barrier permeability and sustain chronic inflammation (23). These preclinical findings suggest that microbial imbalance induces inflammation, while inflammation further disrupts microbial homeostasis, thereby forming a self-reinforcing cycle. Notably, in a mouse study of pancreatic cancer (24), inflammation-induced microbial alterations became evident only in mice carrying Kras mutations and developing advanced pancreatic lesions, including pancreatic intraepithelial neoplasia (PanIN) and pancreatic ductal adenocarcinoma (PDAC). This finding suggests that host genetic background may influence the extent to which microbiota-mediated inflammatory effects are amplified.

At the stage of tumour metastasis, animal studies suggest that microbial structural components are not limited to local inflammation at the primary site. Instead, they may contribute to formation of the pre-metastatic niche through systemic low-grade inflammation. Before tumour cells arrive, the microbiota may help create a microenvironment in distant organs that is suitable for tumour cell survival and growth (25). Circulating LPS and the inflammatory factors it induces can act on the liver, adipose tissue, and vascular wall, triggering local inflammatory responses and amplifying systemic inflammation. They may also induce matrix metalloproteinase-2 (MMP-2) and MMP-9 expression, thereby degrading matrix barriers and facilitating tumour cell infiltration into the vascular wall. In a study of colorectal cancer liver metastasis (26), mice that received oral F. nucleatum showed increased abundance of Enterococcus and Escherichia coli and reduced abundance of Lachnospiraceae. These changes were accompanied by significantly elevated plasma levels of multiple pro-inflammatory cytokines, increased hepatic myeloid-derived suppressor cells (MDSCs) and Tregs, and reduced natural killer (NK) cells and CD8^+^ T cells, together with fewer T helper 17 (Th17) cells. The incidence of colorectal liver metastasis, tumour burden, and liver weight all increased, whereas median survival was shortened. In ESCC (21, 27), increased Bacteroides-related LPS and elevated serum pro-inflammatory cytokines were accompanied by increased expression of the epithelial–mesenchymal transition (EMT) marker vimentin and decreased E-cadherin in tumour tissues. These findings suggest that the inflammatory milieu may promote EMT (28) and thereby facilitate metastasis.

### 2.2 Microbial metabolites mediate inflammation and tumour regulation

In addition to the direct activation of inflammation by microbial structural components, microbial metabolites are important signalling molecules that regulate inflammation and tumours. Microbial metabolism and inflammatory signalling regulate host immunity through multiple pathways and represent a common mechanism that may drive the development of many tumour types.

At the stage of tumour initiation, short-chain fatty acids (SCFAs), including acetate, propionate, and butyrate, are among the most representative candidate anti-inflammatory metabolites generated by intestinal fermentation (29). Current mechanistic studies and animal experiments suggest that they have important roles in regulating inflammation and influencing tumour progression (30). Compared with healthy individuals, patients with gastric cancer show significantly reduced abundance of the key butyrate-producing genera Faecalibacterium and Bifidobacterium, whereas IL-10 expression is significantly increased. Butyrate supplementation downregulates programmed death-ligand 1 (PD-L1) and IL-10 expression and suppresses tumour growth in mice (31). In contrast, agmatine, a metabolite produced by multiple gut bacteria (32), shows pro-inflammatory and pro-tumorigenic effects in colorectal cancer models and patient samples. Agmatine concentrations are significantly increased in the intestines or plasma of colorectal cancer model mice and patients with colorectal cancer (CRC). Agmatine can stabilize β-catenin signalling and is associated with increased IL-6 and TNF-α and decreased IL-10, thereby helping establish a pro-tumorigenic inflammatory microenvironment and promoting colorectal tumorigenesis (33). In addition, indole-3-acetic acid (IAA) can inhibit colorectal tumorigenesis and NSCLC development by promoting IL-35 production or suppressing IL-6 secretion (34, 35). Nisin Z, produced by Lactococcus lactis, can attenuate the intestinal inflammatory milieu and thereby suppress intestinal tumorigenesis (36). Overall, during tumour initiation, SCFAs and some indole derivatives tend to maintain immune homeostasis and limit ineffective inflammation, whereas agmatine and related metabolites are more often associated with accumulation of pro-inflammatory and pro-tumorigenic signals.

At the stage of tumour promotion, gut microbiota-derived metabolites are direct mediators linking dysbiosis to tumour progression. After entering the circulation or accumulating locally, these small metabolites can sustain chronic inflammation and shape a pro-tumorigenic environment through metabolism–inflammation coupling. Indole-3-lactic acid (ILA), a microbial metabolite with anti-cancer properties, can reduce phosphorylated STAT3 (p-STAT3) levels (37). Through regulation of immune homeostasis, it can decrease Th17 cells while increasing Tregs and IL-22-producing group 3 innate lymphoid cells (ILC3s), thereby alleviating inflammation. In contrast, dysregulated bile acid metabolism, especially accumulation of secondary bile acids such as deoxycholic acid (DCA), is more commonly associated with pro-tumorigenic effects. DCA can induce inflammatory cytokine production and upregulate key pro-angiogenic factors such as vascular endothelial growth factor (VEGF) (38, 39), thereby promoting angiogenesis and supporting sustained tumour growth. Thus, different metabolites exert divergent regulatory effects on inflammation and tumours. SCFAs and some indole derivatives tend to maintain homeostasis and alleviate inflammation, whereas agmatine and secondary bile acids are more often associated with amplification of pro-inflammatory and pro-tumorigenic signalling.

At the stage of tumour metastasis, microbial metabolites may also contribute to formation of inflammatory niches in distant organs. After entering the circulation, gut microbiota-derived metabolites can stimulate immune cells to secrete inflammatory factors, which then act on distant organs and alter their immune microenvironment. Factors such as IL-10, TGF-β, and colony-stimulating factor-1 (CSF-1) can promote macrophage polarization toward the M2 phenotype (40–42). These M2 macrophages secrete pro-angiogenic factors and matrix metalloproteinases (43), degrade the extracellular matrix, and provide routes for tumour cell invasion. IL-10 can also induce differentiation of CD4^+^ T cells into Tregs, thereby promoting formation of an immunosuppressive microenvironment (44). Meanwhile, secondary bile acids entering the portal circulation (45) can affect liver sinusoidal endothelial cells and hepatic stellate cells, creating an inflammatory environment that is more permissive for metastasis. DCA and other secondary bile acids can also promote angiogenesis and tumour invasion through activation of multiple pro-metastatic effectors (46), while regulating intestinal barrier formation and inflammation-driven immune dysfunction (47). In addition, indolepropionic acid can influence the expression of EMT-related genes, but it also upregulates E-cadherin and may therefore inhibit EMT (48). In a BALB/c intrasplenic tumour injection model, sodium butyrate reduced IL-10 secretion and increased IL-17 secretion in the liver of mice with colorectal liver metastasis, with a significant reduction in hepatic metastasis (49). These findings suggest that metabolites not only regulate inflammation at the primary tumour site but also influence metastasis-related microenvironmental remodelling.

### 2.3 Microbiota–inflammation interactions remodel the tumour immune microenvironment

In addition to the direct effects of structural components and metabolites, the gut microbiota can also influence the tumour immune microenvironment at the systemic level through sustained interactions with inflammatory networks, as shown in preclinical studies. The main manifestations include innate immune reprogramming, adaptive immune regulation, and establishment of pre-metastatic niches.

At the stage of tumour initiation, under physiological conditions, a balanced gut microbiota helps maintain immune tolerance through its metabolites and cellular components, promotes Treg differentiation and anti-inflammatory cytokine secretion, and creates an immune microenvironment that is less favourable for tumour growth (9). When dysbiosis occurs, however, this balance is disrupted. This disruption can impair local intestinal barrier function and promote systemic inflammatory responses (10) and immune abnormalities (11). Beneficial commensals and their metabolites, especially SCFAs and indole derivatives, can regulate the Treg/Th17 balance and influence secretion of IL-10 and IL-22, thereby maintaining mucosal immune homeostasis. In contrast, pathogenic microbial communities and their inflammatory stimuli may promote tumour initiation through persistent pro-inflammatory cytokine networks.

At the stage of tumour promotion, the chronic inflammatory milieu driven by dysbiosis further alters the composition and function of immune cells and reshapes the TME. On the one hand, microbial imbalance increases intestinal barrier permeability, allowing microbial-associated molecular patterns and bioactive metabolites to continuously enter the circulation. This process induces release of inflammatory cytokines such as IL-6, TNF-α, and IL-1β and promotes chronic inflammation and formation of a pro-tumorigenic niche. On the other hand, these microbial signals can reprogramme local innate immune cells, promote polarization of tumour-associated macrophages toward an M2-like phenotype, enhance recruitment of MDSCs, and weaken dendritic cell antigen presentation, thereby creating an immunosuppressive stroma and pro-angiogenic environment. Prolonged inflammatory stimulation alters immune cell composition, and tumour-associated macrophages gradually shift toward a pro-tumorigenic phenotype, secreting multiple pro-angiogenic and immunosuppressive factors and accelerating tumour growth (50). In a CRC mouse model treated with Streptococcus gallolyticus supernatant (51), IL-17F and IL-22 expression was markedly increased, together with IL-6, IL-1β, IL-8, and cyclooxygenase-2 (COX-2). Expression of the M2 macrophage marker CD206 was also significantly increased in tumour tissues, whereas the M1/M2 macrophage ratio decreased substantially. These findings suggest that the IL-17-related inflammatory axis may participate in M2 macrophage induction and shift tumour-associated macrophages from an anti-tumour to a pro-tumour state. At the same time, beneficial commensal bacteria can enhance dendritic cell maturation and antigen cross-presentation, promote priming, expansion, and effector infiltration of CD8^+^ T cells, and thereby strengthen anti-tumour immunity.

At the stage of tumour metastasis, microbiota–inflammation interactions are no longer confined to the primary site. Instead, they may create conditions that favour tumour cell dissemination and colonization by remodelling the systemic host environment. Inflammatory cytokines can induce matrix degradation, alter vascular permeability, and recruit immunosuppressive cells, thereby facilitating survival and seeding of circulating tumour cells. A study showing that F. nucleatum promotes colorectal cancer liver metastasis demonstrated (26) that this bacterium not only alters gut microbiota composition but also remodels a hepatic immune niche favourable for metastasis by increasing pro-inflammatory cytokine levels and hepatic MDSCs and Tregs while reducing NK cells and CD8^+^ T cells. Meanwhile, microbiota-associated inflammatory environments can also drive EMT and enhance tumour cell invasion and migration (21, 27, 28). Taken together, current mechanistic studies and animal experiments support the view that the gut microbiota is not limited to local inflammatory regulation. Instead, it appears to continuously shape inflammatory intensity, immune composition, and metastatic potential in the TME through key pathways including metabolic signalling, immune cell remodelling, and T-cell priming.

The core mechanisms by which key gut microorganisms and metabolites regulate tumour initiation, promotion, and metastasis through inflammatory signalling networks are summarized in **Table 1**. The regulatory effects of the gut microbiota on intestinal inflammation and the tumour microenvironment are illustrated in **Figure 1**.

**Table 1:** Representative gut microorganisms and metabolites implicated in tumour initiation, promotion, and metastasis through inflammatory cytokine-related mechanisms.

Microorganism or metabolite	Tumour stage	Regulated inflammatory cytokines/signalling pathways	Proposed mechanism of action	Associated tumour type	Reference
*Fusobacterium nucleatum*	Tumour initiation	NF-κB activation; KC ↑, TNF-α ↑, IL-6 ↑, IFN-γ ↑, MCP-1 ↑	Secretes outer membrane vesicles that activate NF-κB-related inflammatory programmes and promote a pro-inflammatory microenvironment	Colorectal cancer	(15)
Butyrate	Tumour initiation	PD-L1 ↓, IL-10 ↓	Butyrate supplementation downregulates PD-L1 and IL-10 expression and is associated with suppressed tumour growth	Gastric cancer	(31)
Agmatine	Tumour initiation	Wnt/β-catenin, *Rnf128*, IL-6 ↑, TNF-α ↑, IL-10 ↓	Binds *Rnf128*, inhibits β-catenin ubiquitination and degradation, activates Wnt signalling, and promotes a pro-inflammatory microenvironment	Colorectal cancer	(33)
IAA	Tumour initiation	IL-35 ↑, IL-6 ↓	Promotes IL-35 production or suppresses IL-6 secretion and is associated with inhibition of tumorigenesis	Colorectal cancer; NSCLC	(34, 35),
Nisin Z (produced by *Lactococcus lactis*)	Tumour initiation	NF-κB ↓	Inhibits NF-κB signalling and attenuates the intestinal inflammatory milieu	Colorectal cancer	(36)
*Bacteroidetes*	Tumour promotion	TLR4/MyD88/NF-κB, IL-6 ↑, TNF-α ↑, TGF-β ↑, IL-10 ↓	LPS enters the circulation, activates TLR4/MyD88 signalling, increases pro-inflammatory cytokines, and suppresses anti-inflammatory cytokines, thereby being associated with ESCC invasion	ESCC	(21)
*Streptococcus gallolyticus*	Tumour promotion	IL-17F ↑, IL-22 ↑, IL-6 ↑, IL-1β ↑, IL-8 ↑, COX-2 ↑, CD206 ↑	Promotes M2 macrophage polarisation and shifts the tumour microenvironment towards a pro-tumour state	Colorectal cancer	(51)
ILA	Tumour promotion	p-STAT3 ↓, AhR, Th17 ↓, Tregs ↑, IL-22 ↑	Reduces p-STAT3, activates AhR-related immune regulation, decreases Th17 cells, and increases Tregs and IL-22-producing ILC3s, thereby helping maintain intestinal homeostasis	Colorectal cancer	(37)
DCA	Tumour promotion	TGR5, STAT3, NF-κB, VEGF ↑	Binds TGR5, induces STAT3 phosphorylation, cooperates with NF-κB-related inflammatory signalling, and upregulates VEGF, thereby promoting angiogenesis	Gastric cancer	(38, 39),
*Fusobacterium nucleatum*	Tumour metastasis	Pro-inflammatory cytokines ↑, MDSCs ↑, Tregs ↑, NK cells ↓, CD8+ T cells ↓, Th17 cells ↓	Oral administration increases immunosuppressive cell populations and reduces effector immune cells, thereby being associated with colorectal liver metastasis	Colorectal cancer liver metastasis	(26)
Secondary bile acids	Tumour metastasis	FXR/TGR5	Influence liver sinusoidal endothelial cells and hepatic stellate cells through FXR/TGR5-related signalling, thereby contributing to an inflammatory environment permissive to metastasis	Colorectal cancer liver metastasis	(45)
Indolepropionic acid	Tumour metastasis	Vimentin, *Fgfbp1*, Snail, β-catenin, E-cadherin ↑	Modulates EMT-related gene expression while upregulating E-cadherin, thereby being associated with EMT inhibition	Colorectal cancer	(48)
Sodium butyrate (NaB)	Tumour metastasis	IL-10 ↓, IL-17 ↑	Reduces IL-10 and increases IL-17 in the liver and is associated with reduced colorectal liver metastasis	Colorectal cancer liver metastasis	(49)

**Figure 1:**
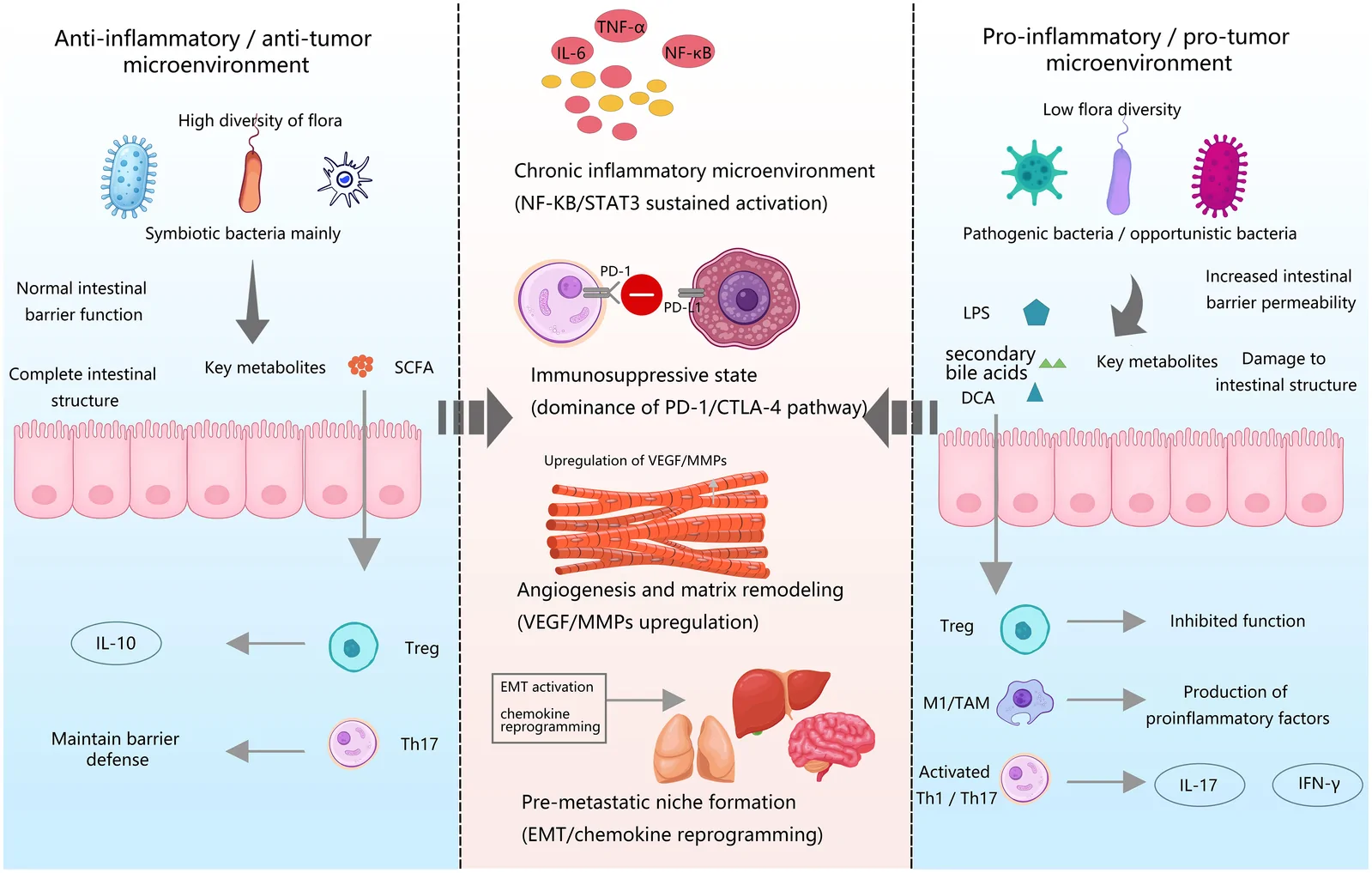
Dual regulatory effects of the gut microbiota on the intestinal inflammatory and tumour microenvironment. Left, regulatory pathways of the anti-inflammatory and anti-tumorigenic intestinal microenvironment. Beneficial commensal gut microbiota maintains the integrity of the intestinal epithelial barrier. Their metabolites, SCFAs, promote the activation and functional activity of Tregs, which in turn markedly upregulate IL-10 expression while downregulating TNF-α and IL-6 levels. This preserves intestinal immune homeostasis and exerts anti-inflammatory and anti-tumorigenic biological effects. Right, regulatory pathways of the pro-inflammatory and pro-tumorigenic intestinal microenvironment. Pathogenic gut bacteria disrupt the intestinal epithelial barrier, releasing pro-inflammatory metabolites and LPS. Pro-inflammatory metabolites inhibit Treg function and downregulate IL-10 expression, while LPS activates the NF-κB signalling pathway via TLR4, markedly upregulating the expression of the pro-inflammatory cytokines TNF-α and IL-6. Ultimately, this mediates the progression of chronic intestinal inflammation and the formation of a pro-tumorigenic microenvironment.

## 3 Roles and mechanisms of the gut microbiota in cancer therapy

In recent years, many basic and clinical studies have shown that the gut microbiota is involved not only in tumour development but also in the efficacy and adverse effects of multiple anti-cancer therapies. A central mechanism involves regulation of local and systemic inflammatory balance, with inflammatory cytokines acting as key signalling molecules that affect treatment-related pathophysiological processes.

### 3.1 Role of the gut microbiota in chemotherapy

Preclinical studies and some patient cohort studies indicate that chemotherapy can alter both the gut microbiota and inflammatory cytokines. While eliminating tumour cells, chemotherapeutic agents often cause intestinal epithelial injury and microbial dysbiosis (52). These effects can increase intestinal permeability and facilitate microbial translocation into the mucosa and circulation, thereby triggering or aggravating systemic inflammatory responses. At the same time, chemotherapy-induced neutropenia weakens intestinal immune defence, increases inflammatory cytokine levels, and further impairs barrier function (53). Dysbiotic microbiota and elevated inflammatory cytokines may then reinforce each other, collectively exacerbating chemotherapy-related intestinal toxicity. Studies have shown that chemotherapy directly alters gut microbial community structure in cancer patients while also increasing circulating TNF-α levels (54, 55). Cisplatin can disrupt expression of intestinal epithelial tight junction proteins such as zonula occludens-1 (ZO-1), occludin, and claudin-5, thereby increasing intestinal permeability and promoting translocation of bacterial components and endotoxins, which in turn activates intestinal and systemic inflammatory responses (56). Notably, another study showed that oxaliplatin exerted anti-inflammatory effects in a rat model of colorectal cancer. After oxaliplatin treatment, serum TNF-α, C-reactive protein (CRP), and IL-1β levels were significantly reduced, IL-4 was significantly increased, and the SCFA-producing genus Alistipes was enriched (57).

Changes in the gut microbiota and inflammatory cytokines can also affect chemotherapy efficacy and toxicity (58). In cell experiments, infection of CRC cells with F. nucleatum activated TLR4 and triggered persistent downstream NF-κB signalling. This activation caused CRC cells to resist 5-fluorouracil (5-FU)-induced apoptosis and reduced chemosensitivity. In patients with advanced CRC, high F. nucleatum abundance was associated with significantly increased chemoresistance (59).

### 3.2 Role of the gut microbiota in immunotherapy

The efficacy of immunotherapy depends strongly on the activation status of the immune system, and the gut microbiota can directly influence therapeutic outcomes by shaping the inflammatory microenvironment. When gut microbial homeostasis is disrupted, inflammatory responses often become chronic and ineffective, leading to immune exhaustion and activation of immunosuppressive inflammatory pathways, which may reduce the clinical benefit of immunotherapy. By modulating the intensity of inflammatory responses, the gut microbiota can exert a double-edged effect on both efficacy and toxicity.

The efficacy of ICIs is closely associated with microbiota status (60). Multiple observational clinical studies have found that the abundance of specific microbial taxa is significantly associated with ICI response (61, 62). Microbes such as Akkermansia muciniphila, Faecalibacterium prausnitzii, and Bifidobacterium spp. are enriched in ICI responders. In patients with NSCLC and melanoma, higher abundance of A. muciniphila is associated with higher objective response rates (ORR) and longer overall survival (OS) (63).

Current mechanistic studies and clinical association analyses suggest that the microbiota influences ICI efficacy by regulating inflammatory cytokine networks. Products associated with Gram-negative bacteria such as Bacteroides and Prevotella can induce local and systemic pro-inflammatory responses, creating a chronic but ineffective inflammatory state that may ultimately lead to poor response to programmed cell death protein 1 (PD-1) inhibitors (64). Clinical data further show (65) that the abundance of the genus Alistipes is significantly associated with attenuated response to neoadjuvant immunotherapy in patients with lung adenocarcinoma (LUAD). IL-6, as a key inflammatory cytokine, may mediate signalling from intestinal inflammation to pulmonary tumour progression and immunosuppression.

### 3.3 Immune-related adverse events

Immune-related adverse events (irAEs) are a major factor that limits ICI therapy, and the gut microbiota may have protective or promotive roles in their development (66). Importantly, discussions of beneficial microbiota in anti-tumour immunity should also consider potential risks and trade-offs. Although many studies emphasize associations between commensals such as Akkermansia, Faecalibacterium, and Bifidobacterium and immunotherapy benefit, excessive immune activation may also increase the risk of irAEs. Current clinical observations suggest that certain microbial signatures may be associated not only with stronger anti-tumour immunity but also with higher risks of colitis, dermatitis, or hepatitis. A reduction in the family Ruminococcaceae is associated with severe irAEs (67). This family includes F. prausnitzii, a well-known anti-inflammatory bacterium (68). Other clinical observations suggest that patients who develop severe colitis-related irAEs typically have lower abundance of F. prausnitzii (69), whereas Bifidobacterium may reduce inflammatory responses and mitigate immunotoxicity by modulating inflammatory cytokine levels (70).

Taken together, the benefit of the microbiota does not simply depend on enhancing immunity. Rather, it lies in achieving a balance between effective anti-tumour responses and controllable inflammatory toxicity. Therefore, future studies on microbiota-based interventions should evaluate not only response rates but also the incidence, severity, organ specificity, and long-term immune sequelae of irAEs.

### 3.4 Gut microbiota stratification, heterogeneity of immunotherapy response, and potential for precision medicine

Although substantial evidence indicates that the gut microbiota can influence the efficacy of chemotherapy and ICIs, growing data suggest that this effect is not determined by a single microorganism. Patients differ markedly in baseline microbiota composition, metabolic functional modules, inflammatory cytokine profiles, and host immune thresholds. These differences may jointly influence immunotherapy sensitivity, resistance, and risk of irAEs.

#### 3.4.1 Associations between different gut microbiota subgroups and immunotherapy response

Current studies show that responders and non-responders to ICIs often display different microbiota enrichment patterns. In general, subgroups enriched in commensals such as A. muciniphila, F. prausnitzii, and Bifidobacterium spp (61). are often associated with stronger antigen presentation, higher CD8^+^ T-cell infiltration, and better ORR and OS. These microbiota may promote dendritic cell maturation, enhance cross-presentation, improve T-cell priming, and regulate inflammatory intensity through SCFAs, indole metabolites, and related mediators, thereby helping maintain the TME in an anti-tumour immune state (37). In contrast, subgroups dominated by Gram-negative opportunistic pathogens, LPS-high communities, or pro-inflammatory metabolic modules are more frequently observed in patients with poor immunotherapy response (64). These dysbiotic states are often accompanied by persistent elevation of IL-1β, IL-6, TNF-α, C-X-C motif chemokine ligand 8 (CXCL8), and other pro-inflammatory mediators. This condition can lead to chronic but ineffective systemic inflammation, T-cell exhaustion, recruitment of MDSCs, and polarization of tumour-associated macrophages toward an M2-like phenotype, thereby weakening the efficacy of PD-1/programmed death-ligand 1 (PD-L1) blockade (65). It should be noted that these conclusions are based mainly on observational studies and mechanistic inference. The dominant beneficial taxa are not fully consistent across tumour types, suggesting that so-called beneficial bacteria are context-dependent and influenced by tumour type, host background, and ecological niche.

#### 3.4.2 Potential roles of the gut microbiota in precision medicine

From a translational perspective, the gut microbiota and related inflammatory cytokine networks may have important roles in precision oncology. First, they may serve as predictive biomarkers to identify patients who are more likely to benefit from ICIs. By integrating faecal metagenomics, microbial metabolite profiles, serum inflammatory cytokines, and tumour immune infiltration features, it may be possible to develop prediction models that are more comprehensive than those based only on PD-L1 expression or tumour mutational burden (TMB) (71). Second, they may serve as toxicity stratification tools to identify patients at higher risk of irAEs (72). Loss of certain anti-inflammatory commensals, expansion of inflammation-associated microbes, and elevated baseline inflammatory cytokines may indicate a high-risk immune toxicity state. Third, microbiota features may help guide therapeutic decision-making and support individualized intervention strategies designed to improve the therapeutic window (73).

#### 3.4.3 How to enhance immunotherapy response under different microbiota backgrounds

Improving immunotherapy response does not simply require increasing beneficial bacteria. A more important goal is to reconstruct a microbiota–inflammation balance that supports anti-tumour immunity under specific host conditions. Potential strategies for different microbiota subgroups include the following.

First, microbiota remodelling strategies may be considered. For patients with reduced microbial diversity and depletion of commensals, high-fibre diets, prebiotics, specific probiotic formulations, or postbiotic supplementation may help promote production of SCFAs and indole metabolites. These interventions may enhance dendritic cell function, improve CD8^+^ T-cell priming, and reduce ineffective chronic inflammation (74). For patients with severe dysbiosis, faecal microbiota transplantation (FMT) may provide a stronger means of microbial reconstruction, especially in patients with primary or secondary resistance to anti-PD-1 therapy, where it has shown translational potential (75).

Second, disruptive exposures should be minimized where possible. Existing studies suggest that broad-spectrum antibiotics, inappropriate use of acid-suppressive agents, unhealthy dietary patterns, and long-term nutritional imbalance may impair microbial homeostasis and reduce ICI efficacy (76). Therefore, throughout cancer treatment, the timing and indication for antibiotic use should be optimized whenever possible, unnecessary microbiota disruption should be avoided, and gut ecological stability should be supported through nutritional management.

Third, combined modulation of inflammatory pathways may be useful in selected settings. For patients with inflammation-driven poor response characterized by high IL-6, TNF-α, or persistent LPS burden, future studies may explore combining microbiota-targeted interventions with inflammatory cytokine modulation to interrupt the cycle of dysbiosis, chronic inflammation, and immunosuppression. The aim of this strategy is not simply to suppress inflammation, but to reshape ineffective, exhaustion-associated chronic inflammation into an immune-activated state that is more favourable for effector T-cell function (77).

Finally, dynamic monitoring and individualized adjustment are important. The microbiota is not static. It changes before treatment, during treatment, and throughout disease progression. Precision medicine should therefore not rely only on a single faecal test. Instead, it should combine longitudinal microbiota monitoring, dynamic inflammatory cytokine changes, radiological response assessment, and irAE occurrence to guide timely adjustment of microecological intervention strategies. Only by incorporating microbiota characteristics into a dynamic therapeutic management framework can their clinical utility be more fully realized.

#### 3.4.4 Summary

Overall, the potential value of the gut microbiota in precision medicine lies not only in its use as a predictive indicator of immunotherapy response and toxicity, but also in its modifiability. Different gut microbiota subgroups influence inflammatory cytokine networks, innate immune reprogramming, and T-cell activation, thereby contributing to differential ICI outcomes. If a standardized and reproducible microbiota stratification system can be established and integrated with metabolite profiles, inflammatory cytokine signatures, and clinical characteristics, it may help identify patients most likely to benefit from immunotherapy and provide a theoretical basis for microbiota-targeted combination strategies that enhance efficacy and reduce toxicity. The gut microbiota can modulate the efficacy and toxicity of chemotherapy and immunotherapy by affecting inflammatory cytokine networks, intestinal barrier integrity, and anti-tumour immune status. Interventions targeting these key pathological processes may provide promising translational approaches (**Figure 2**). Major related mechanisms and evidence types are summarized in **Table 2**.

**Figure 2:**
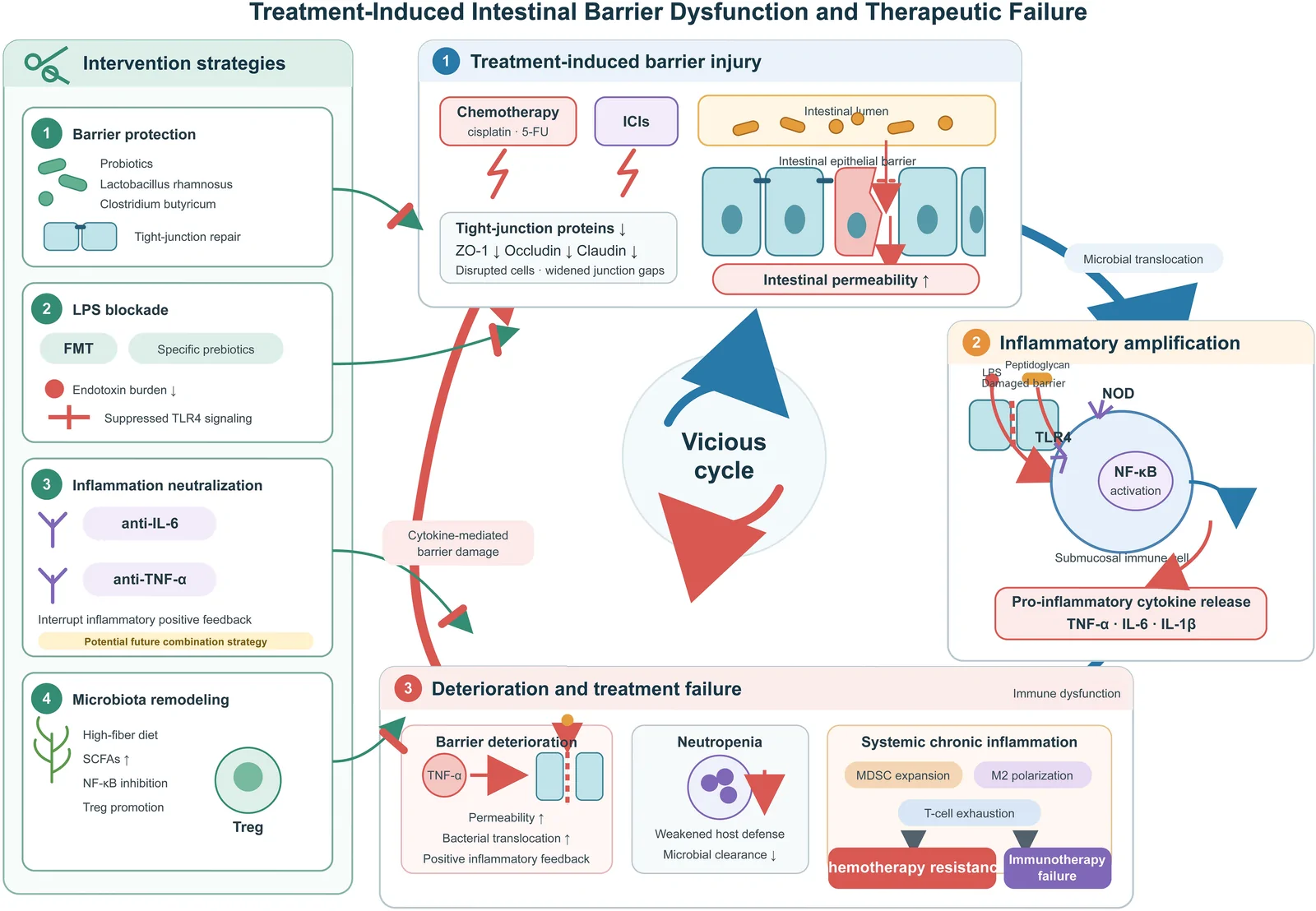
Schematic illustration of treatment-induced intestinal barrier dysfunction, inflammatory amplification, and therapeutic failure. This figure illustrates how chemotherapy and immune checkpoint inhibitors can damage the intestinal epithelial barrier, reduce tight-junction protein expression, and increase intestinal permeability, thereby promoting microbial translocation and inflammatory signalling. The resulting pro-inflammatory cytokine release further exacerbates barrier disruption and immune dysfunction, creating a vicious cycle that ultimately contributes to chemotherapy resistance and immunotherapy failure. Potential intervention strategies, including barrier protection, LPS blockade, inflammation neutralization, and microbiota remodelling, are also summarized.

**Table 2:** Representative evidence on how the gut microbiota may influence anti-cancer therapy efficacy and toxicity through inflammatory cytokines and related pathways.

Treatment modality	Microbiota-related changes	Key inflammatory cytokines/signalling pathways	Potential effects on efficacy	Potential effects on toxicity	Evidence type	Representative references
Chemotherapy	Chemotherapy may damage the intestinal epithelial barrier and disturb microbial homeostasis, leading to dysbiosis, increased intestinal permeability, and translocation of bacterial components	TNF-α, IL-1β, IL-6, TLR4/NF-κB; ZO-1, occludin, claudin-5	Dysbiosis may reduce chemosensitivity and has been associated with resistance to some agents	May aggravate mucosal injury, intestinal inflammation, and systemic inflammatory responses, thereby increasing treatment-related toxicity	Animal experiments; observational clinical studies	(52–56)
5-FU	Increased abundance of *Fusobacterium nucleatum* and interaction with CRC cells	TLR4/NF-κB	Persistent activation of pro-inflammatory signalling suppresses tumour-cell apoptosis and is associated with 5-FU resistance	May exacerbate tissue injury by sustaining a local pro-inflammatory state	Cell experiments; observational clinical studies	(59)
Oxaliplatin	May remodel gut microbiota composition and enrich SCFA-related genera such as *Alistipes*	TNF-α ↓, CRP ↓, IL-1β ↓, IL-4 ↑	May indirectly enhance therapeutic benefit by improving inflammatory status and microbial ecology	May reduce inflammation-associated adverse effects	Animal experiments	(57)
ICIs	Responders are often enriched in commensals such as *Akkermansia muciniphila*, *Faecalibacterium prausnitzii*, and *Bifidobacterium* spp., whereas poor responders more often show pro-inflammatory dysbiosis	IL-6, TNF-α, IFN-γ; antigen presentation- and CD8+ T-cell activation-related pathways	Beneficial microbiota are associated with higher response rates and longer survival; pro-inflammatory dysbiosis may weaken ICI efficacy	Enhanced immune activation may be accompanied by increased risk of immune toxicity	Observational clinical studies; mechanistic studies	(61–65)
irAEs	Reduced *Ruminococcaceae*, lower *F. prausnitzii* abundance, and impaired inflammation-suppressive capacity	Cytokine imbalance and pathways associated with excessive immune activation	May coexist with enhanced anti-tumour immunity, suggesting a possible coupling between efficacy and toxicity	Associated with increased risks of colitis, dermatitis, and hepatitis; some *Bifidobacterium* strains may be protective	Observational clinical studies; animal experiments	(66–70)
FMT	Healthy-donor FMT may increase beneficial taxa and reduce pathogenic colonisation, whereas different donor sources may produce heterogeneous effects	TNF-α/COX-2, Th1/Th17, EMT, Wnt/β-catenin-related pathways	May restore a microbiota structure favourable to anti-tumour immunity, overcome immunotherapy resistance, and enhance therapeutic response	Inappropriate donor sources may aggravate inflammatory imbalance or promote tumour progression; infectious transmission remains a concern	Animal experiments; early clinical studies	(75, 78, 79)
Probiotics, prebiotics, and dietary interventions	May increase beneficial commensals and SCFA production and improve microbial diversity and barrier function	TNF-α ↓, IL-6 ↓, TLR4/NF-κB inhibition; some studies report IL-2 ↑ and IFN-γ ↑	May improve the gut microecological environment and enhance anti-tumour immunity	May alleviate chronic inflammation and reduce intestinal toxicity and immune imbalance	Animal experiments; mechanistic studies; some clinical studies	(80–83)

## 4 Cancer interventions based on gut microbiota and inflammation

With the in-depth understanding of the mechanism of microbiota-inflammation interaction, intervention strategies that regulate the structure of the microbiota are becoming an emerging direction for cancer treatment, and the development of new strategies to regulate the structure of the microbiota has become a hot topic in cancer therapy, these strategies are able to re-establish intestinal homeostasis, alleviate inflammatory responses, and enhance anti-tumor immunity, which may provide new insights into the treatment of cancer, it mainly includes probiotics and prebiotics intervention, dietary regulation, antibiotics and targeted inflammation therapy, fecal microbiota transplantation (FMT).

### 4.1 Probiotic interventions

Probiotics have been widely studied for restoring microbial homeostasis and improving the inflammatory milieu, although current anti-tumour evidence mainly comes from animal experiments and *in vitro* studies. In CRC, local colonic inflammation was markedly alleviated in mice treated with Bifidobacterium, and tumour progression was suppressed through inhibition of the inflammatory state (80). Interventions using Clostridium butyricum, A. muciniphila (81), and Lactiplantibacillus plantarum-12 (82) significantly reduced levels of pro-inflammatory cytokines such as TNF-α, IL-1α, and IL-6 in serum and tissues by modulating immune and inflammatory cells within the TME, thereby inhibiting tumour progression. Lacticaseibacillus rhamnosus attenuated colonic inflammation by inhibiting the TLR4/NF-κB pathway and overexpression of pro-inflammatory cytokines and chemokines, thereby suppressing colitis-associated CRC (84). Probiotic therapy may also attenuate inflammation and tumorigenesis through inhibition of the TLR4/NF-κB pathway (85). In addition, Companilactobacillus crustorum MN047 and Lactobacillus coryniformis MXJ32 can improve intestinal barrier integrity (86). After treatment with Probio-M9, expression of p-STAT3 and phosphorylated protein kinase B (p-Akt) in tumour tissues was significantly inhibited, the increase in M1 macrophages was suppressed, and the abundance of beneficial bacteria such as Akkermansia, Bifidobacterium, and Lactobacillus increased (87).

### 4.2 Prebiotic interventions

Prebiotics are food ingredients that cannot be digested or absorbed by the human host but can selectively promote growth of beneficial intestinal bacteria, thereby improving host health (88). They may reduce inflammation and inhibit carcinogenesis by modulating the gut microbiota. Lactulose, a galactose–fructose disaccharide, is a classic prebiotic (89). Studies have shown (83) that lactulose intervention restores gut microbiota balance, reduces pathogen abundance, suppresses TNF-α and IL-6 expression, reduces infiltration of pro-inflammatory macrophages, and ultimately inhibits inflammation-associated tumorigenesis. Cranberry proanthocyanidins can reshape the gut microbiome and oesophageal metabolome, inhibit the TLR/NF-κB/tumour protein p53 (TP53) pathway, reduce multiple pro-inflammatory cytokines and chemokines, and thereby suppress progression of oesophageal adenocarcinoma (90).

### 4.3 Faecal microbiota transplantation

FMT is a powerful strategy for reconstructing gut microbial diversity and metabolic function and has shown translational potential in cancer immunotherapy in recent years. By transplanting gut microbiota from healthy donors, FMT can restore microbial diversity, reduce colonization by harmful bacteria, and improve intestinal barrier function. This process may inhibit release of inflammatory cytokines, reduce chronic inflammatory states, and thereby lower the risk of tumorigenesis. Studies have shown (78) that healthy-donor FMT increases the abundance of Akkermansia, Faecalibacterium, and Ileibacterium while reducing Fusobacterium. These changes inhibit the TNF-α/COX-2 inflammatory pathway, block EMT and Wnt/β-catenin oncogenic signalling, and reduce tumour burden and immune-inflammatory responses. By contrast, FMT from patients with CRC aggravated tumour progression. FMT can also serve as a vehicle to transfer favourable microbiota shaped by combined ginseng polysaccharide and PD-1 antibody treatment to recipients (79).

Importantly, a colorectal cancer study showed (78) that the impact of FMT on tumour therapy depends strongly on donor source. FMT from different sources exerted distinct regulatory effects on the microbiota of CRC mice and resulted in opposite therapeutic outcomes. After healthy control-derived FMT, the abundance of Lactobacillus, Akkermansia, and Ileibacterium in the murine gut increased, whereas mice receiving CRC-derived FMT showed the most aggressive phenotype. The mechanisms underlying these differential effects were mainly associated with the numbers of T helper 1 (Th1) and Th17 cells and the expression of EMT markers and the Wnt/β-catenin pathway.

### 4.4 Dietary and lifestyle interventions

Dietary structure is an important exogenous factor that influences microbiota composition and inflammatory status (91). Dietary interventions targeting the gut microbiome are relatively safe, economical, and feasible strategies for secondary prevention and risk management of cancer. In a study of patients at high risk of colorectal neoplasia, daily consumption of legumes modulated the gut microbiota and its metabolites, affected specific inflammatory and immune response markers, and showed potential anti-cancer effects (74). Diet influences gut microbiota composition, regulates SCFA production, subsequently affects inflammatory cytokine generation, and may influence tumour immunotherapy outcomes (73). High-fibre, low-fat diets can increase butyrate concentrations (92), inhibit the NF-κB pathway, and alleviate chronic inflammation (93).

Probiotic fermented milk is a dairy product that contains live probiotic microorganisms produced through microbial fermentation. It has properties that include modulation of the gut microbiota, relief of constipation, and enhancement of immunity (94). BX-245 fermented milk (95) not only modulates the gut microbiota but also directly regulates the intratumoural microbiota, significantly reduces intestinal barrier permeability, and alleviates barrier damage. After administration of BX-245 fermented milk, serum IL-2 and IFN-γ levels in mice were significantly increased and tumour burden was reduced.

In addition, studies indicate that regular exercise (96) can improve inflammatory status by reducing systemic inflammatory markers, indirectly regulate gut microbiota balance, and enhance the efficacy of ICIs in melanoma (97).

## 5 Perspectives and conclusions

### 5.1 Microbiota–inflammation interactions as a core regulatory hub in cancer

A growing body of evidence indicates that interactions between the gut microbiota and inflammatory cytokines form an important regulatory axis that coordinates tumour initiation, progression, metastasis, and therapeutic response. The gut microbiota is not merely a passive bystander. It dynamically interacts with the host immune system, epithelial barrier, and metabolic networks through microbial structural components and bioactive metabolites, thereby regulating inflammatory signalling cascades at multiple levels. Dysbiosis can induce chronic low-grade inflammation through multiple interconnected inflammatory and immune pathways, driving the TME toward immunosuppression, angiogenesis, and EMT. At the same time, inflammatory cytokine networks may feedback to reshape microbial ecology and metabolic output, creating a continuously amplifying feed-forward loop.

### 5.2 Limitations of current evidence

However, current evidence remains largely descriptive and association-based, and inconsistencies across studies suggest that the relationships among the microbiota, inflammation, and tumours should be interpreted cautiously.

Many mechanistic conclusions rely on germ-free animal models, antibiotic-depletion models, faecal transplantation models, and *in vitro* co-culture systems. Although these models are highly valuable for identifying potential pathways, their microbial composition, immune background, housing environment, and tumour induction methods differ substantially from those in humans. Therefore, their findings cannot necessarily be directly extrapolated to clinical populations. Human studies have greater clinical relevance, but most are cross-sectional studies, retrospective cohorts, or prospective observational studies with limited sample sizes. As a result, they often show association rather than causality. At present, whether specific microbial taxa or metabolites directly drive tumour occurrence and progression, or instead represent accompanying markers of disease state, still requires confirmation through higher-quality evidence.

In addition, microbiota changes observed in clinical studies are not necessarily caused by a single microorganism. Even when certain taxa are enriched in immunotherapy responders, this does not by itself establish a causal role in enhancing efficacy. Similarly, some metabolites may exert bidirectional or even opposite effects depending on tumour type and host context. Thus, the effects of the microbiota are clearly niche-dependent, dose-dependent, and host-dependent.

The biological significance of inflammatory cytokines is also complex and can appear contradictory across studies, especially for classical immunoregulatory cytokines such as IL-10 and TGF-β. Their roles should not be reduced to simple assumptions such as anti-inflammatory meaning anti-tumour or immunosuppressive meaning pro-tumour. Rather, their functions depend strongly on timing, tissue, cell source, and disease stage. For example, IL-10 may exert protective effects in early tumour development or inflammation-driven carcinogenesis by suppressing excessive inflammation, limiting tissue injury, and maintaining barrier homeostasis. However, within an established TME, IL-10 may also promote immune suppression, support Treg function, weaken antigen presentation and effector T-cell activity, and thereby facilitate tumour immune escape. Similarly, TGF-β may suppress abnormal epithelial proliferation and play an anti-tumour role in normal tissue homeostasis and early carcinogenesis, but during tumour progression it often shifts toward a pro-tumour role by promoting EMT, stromal remodelling, angiogenesis, and immune exclusion.

Therefore, opposite conclusions regarding the same cytokine do not necessarily indicate poor study quality. Instead, they may reflect differences in experimental models, tumour types, disease stages, local microenvironment composition, and timing of measurement. Ignoring this context dependence and interpreting IL-10, TGF-β, and related factors in a unidirectional way risks oversimplifying microbiota–inflammation–tumour interactions. Future studies should place greater emphasis on the dynamic trajectories of cytokine changes, the lineage of source cells, and their coupling with specific microbial metabolites in order to define their stage-specific roles in tumour initiation, promotion, metastasis, and therapeutic response more accurately.

Furthermore, much of the current literature remains preclinical and may not translate into stable clinical benefit. In immunotherapy, evidence that the microbiome promotes ICI response has attracted considerable attention, but findings are not fully consistent across cohorts and cancer types. The list of favourable taxa is clearly heterogeneous, and some studies have failed to reproduce earlier results. This pattern suggests that the impact of the gut microbiota on therapeutic efficacy may not be determined by a single species, but rather by the combined effects of microbial network structure, metabolic functional modules, and host immune thresholds.

Microbiome research itself also has methodological limitations. Differences in sampling time points, faecal storage conditions, DNA extraction procedures, sequencing platforms, bioinformatics pipelines, and statistical models limit reproducibility across studies. Moreover, 16S ribosomal RNA (16S rRNA) sequencing provides only limited taxonomic information and cannot accurately capture strain-level differences and functional heterogeneity. Even when metagenomics, transcriptomics, or metabolomics are used, batch effects, difficulties in data integration, and incomplete causal chains remain major challenges. Finally, cancer populations are highly heterogeneous. Tumour type, stage, line of therapy, prior medication history, and nutritional status can all substantially affect microbiota composition, making comparison across studies difficult.

### 5.3 Challenges faced by intervention strategies

At the level of clinical translation, microbiome-based interventions show promise but still face multiple practical challenges. The first challenge is regulation. Unlike conventional small-molecule drugs, probiotics, FMT, and engineered bacterial therapies still differ in product definition, quality control, batch consistency, long-term follow-up requirements, and approval pathways. Their regulatory classification, donor screening standards, pathogen detection scope, and manufacturing specifications have not yet been fully standardized.

The second challenge is safety. Although FMT has unique advantages in restoring microbial diversity, potential risks include pathogen transmission, transfer of antibiotic resistance genes, opportunistic infection, bacteraemia, metabolic abnormalities, and unpredictable immune consequences in certain hosts. These risks require especially careful evaluation in cancer patients receiving chemotherapy or immunotherapy, or in those with impaired haematopoietic function. Similarly, the safety windows for different strains, doses, schedules, and combination regimens remain insufficiently defined.

Current clinical trial design also limits generalizability. Many studies are small, single-centre, and include heterogeneous populations. In addition, microbiota changes are often treated as exploratory rather than prespecified primary endpoints. Cancer treatment itself is highly dynamic, and patients often receive antibiotics, steroids, nutritional support, and other concomitant therapies during treatment. These factors may confound the true effects of microbiota interventions.

Another urgent issue is standardization. At present, microbiome-based therapies still lack unified standards. Even within FMT, donor source, processing method, cold-chain storage, and transplantation frequency can all significantly affect efficacy. For probiotics and postbiotics, strain specificity determines functional differences. Therefore, findings from one strain or genus should not be generalized directly to all related products.

### 5.4 Future directions and concluding remarks

Overall, interactions between the gut microbiota and inflammatory cytokines represent a highly promising but still developing field in oncology. Existing studies provide substantial biological plausibility for their involvement in tumour development and treatment response, but considerable work remains before they can be translated into mature, reproducible, regulated, and broadly applicable clinical interventions. Future research should move beyond simply identifying associated microbes and instead focus on validating causal mechanisms and establishing clinically actionable pathways. First, large-scale, multicentre, longitudinal human studies should be conducted with refined stratification according to diet, drug exposure, and host immune background. Second, germ-free animals, humanized microbiota models, and multi-omics integration strategies should be used to strengthen closed-loop validation between mechanistic studies and clinical observations. Third, safety, standardization, and regulatory feasibility should be emphasized to support development of FMT, probiotics, engineered bacteria, and metabolite-based interventions within more rigorous clinical frameworks. Fourth, composite biomarker systems that integrate microbiota features, metabolite profiles, and inflammatory cytokine networks should be developed to predict efficacy, toxicity, and beneficiary populations.

In conclusion, the gut microbiota–inflammatory cytokine axis provides a new theoretical framework for understanding tumour biology and optimizing therapeutic strategies. However, its incorporation into precision oncology practice still depends on more rigorous causal inference, higher-quality human evidence, and more complete standardization and regulatory systems. Only by fully recognizing its complexity, uncertainty, and potential risks can microbiome-based intervention evolve from a promising concept into a safe, effective, and implementable clinical tool.
